# The relationship between objectively-measured attributes of the built environment and selected cardiovascular risk factors in a South African urban setting

**DOI:** 10.1186/s12889-018-5772-3

**Published:** 2018-07-09

**Authors:** Pasmore Malambo, Anniza De Villiers, Estelle V. Lambert, Thandi Puoane, Andre P. Kengne

**Affiliations:** 1 Faculty of Community and Health Sciences, School of Public Health, University of Western Cape, Robert Sobukwe Rd, Bellville, Cape Town, 7535 South Africa; 2 South African Medical Research Council, Francie van Zijl Drive, Parowvallei, P.O. Box 19070, Cape Town, Tygerberg 7505 South Africa; 3 Division of Exercise Science and Sports Medicine, Department of Human Biology, Faculty of Health Sciences, University of Cape Town, P.O. Box 115, Cape Town, Newlands 7725 South Africa

**Keywords:** Built environment, Walkability, Physical activity, Geographic information system, Accelerometer, Objective measurement, Body mass index, Blood pressure, Risk factors, South Africa

## Abstract

**Background:**

Evidence concerning the relationship between objectively-measured attributes of the built environment with cardio-metabolic risk in populations from lower- and middle-income countries is lacking. In this paper, we describe the association between the objectively-measured built environment with body mass index, blood pressure and physical activity in adult South Africans.

**Methods:**

This cross-sectional study included 341 adults aged ≥35 years drawn from the Cape Town arm of the Prospective Urban Rural Epidemiology (PURE) cohort study. All Cape Town PURE participants were invited to take part in the study. Actigraph GT3X accelerometer and Geographic Information Systems were used to measure physical activity and built environment attributes (community center, shopping center and taxi rank).

**Results:**

In age and sex adjusted models (reference 500 m), access to community centers (1000 m) was positively related to body mass index [beta 4.70 (95%CI: 2.06 to 7.34)] and diastolic blood pressure [4.97 (0.00 to 9.95)]. Distance from a community center (1600 m) was positively related to diastolic blood pressure [6.58 (1.57 to 11.58)] and inversely with moderate-to-vigorous physical activity [− 69.30 (− 134.92 to − 3.70)]. Distance to a shopping center (1600 m) was positively related to body mass index [4.78 (1.11 to 8.45)] and shopping center (1000 m) was positively related to systolic blood pressure respectively [76.99 (0.03 to 83.95)].

**Conclusion:**

Distance to community and shopping centers were significantly associated with BMI, systolic, diastolic blood pressure and moderate-to-vigorous physical activity. Future research should include multiple aspects of built environment variables in order to provide for a broader understanding of their effect on cardiovascular risk profile of African populations.

## Background

Non-communicable diseases, such as cardiovascular disease (CVD), are the leading cause of death in high income and many low- and middle income countries (LMICs) [1], including South Africa [2]. Rises in CVDs diagnoses are attributable to obesity, high blood pressure and physical inactivity [3] compounded by a change in lifestyle behaviors [4]. These rising figures of CVDs are of concern in South Africa and other countries in the region [5].

Rapid epidemiological shifts in population health and disease burden have been linked to changes in the built environment that may promote or adversely affect active transport, such as walking to and from places, because this has been associated with reduced risk of obesity, and non-communicable diseases (NCDs) [6–8]. A recent research indicates that high levels of physical activity may attenuate the negative health burden such as obesity [9]. However, the majority of studies concerning objective and subjective measures of the built environment, including attributes of walkability such as land use mix and proximity to home, street or network connectivity, population density, pedestrian infrastructure, aesthetics, and safety, have been undertaken in developed or high-income countries [10–12], where the context, culture and perceptions regarding access may be specific [13]. At least one study in a middle-income country has linked aspects of the neighborhood and built environment such as: water supply, garbage collection and street lighting to health status in older adults, [14]. Nevertheless, both objective and subjective studies incorporating measures of the built environment are sparse [15] and those studies that have used them have shown mixed results in both low-income [16] and high-income countries [17]. Thus, it is unknown whether one measurement method is more effective than the other for assessing the neighborhood environment and its association with obesity, body mass index (BMI), hypertension and physical activity especially among South African adults.

Built environments that support utilitarian walking, with destinations in close proximity, have been linked to lower body mass index (BMI) [18], blood pressures (BP) [19] and more walking for transport [20]. Access to a mix of recreational and non-recreational destinations such as schools, coffee shops, supermarkets, other retail shops and services within the residential neighborhood, has been reported to be positively related with walking for transportation and leisure [21, 22]. However, it has been suggested that lack of facilities for pedestrians, perceived neighborhood safety, less accessible destinations, and poorly-connected street patterns, were all associated with reduced physical activity and obesity risk. A study in the USA found that the absence of nearby nonresidential destinations, lack of footpaths or poor quality when available, disagreeable community, inexistent interesting sites, presence of waste and physical disorders were significantly associated with obesity [23]. Other studies have associated a walkable environment with walking, bicycling, using public transit, and a decreased likelihood for driving or owning a car [24]. Studies have also linked a high availability of fast-food outlets relative to conventional restaurants in a neighborhood environment, with higher and lower BMI, respectively [25]. In addition, a recent systematic review found most built environment attributes to be related with cardiovascular disease risk factors such as BMI, PA, hypertension, and type 2 diabetes [26].

Given that cardiovascular disease in South Africa and Africa at large is increasing [5], developing and implementing built environment strategies that promote physical activity and thus reduce CVD risk, requires input from context-specific studies. Yet, there is little empirical evidence that has used objectively-measured attributes of the built environment with CVD risk factors in an urban African context. Therefore, the aim of this study was to evaluate the relationship between objectively-measured built environmental attributes with BMI, systolic BP (SBP), diastolic BP (DBP) and physical activity in adult South Africans.

## Methods

### Study setting and population

Participants in the current study were drawn from the Langa Township site of the Prospective Urban Rural Epidemiology (PURE) cohort study in Cape Town, South Africa. The cohort, established in 2009, included 2064 Black South African men and women aged 35–70 years. In this study, all PURE participants were invited to take part in the study. Included participants had to: (1) be aged 35–70 years, (2) be living within identified household, and (3) have no disability that precluded walking.

### Study design and sample size

This was a cross-sectional survey nested within PURE follow-up evaluation conducted in 2013. Addressee’s of all the Langa PURE cohort participants (*N* = 459) with valid anthropometric measurements were used for geocoding. We merged each participant’s accelerometer data with the corresponding GIS data according to their ID numbers, so that each Geographic Information System (GIS) point had a corresponding accelerometer count. However, only a representative sample of 341 individuals, both men and women, with valid data on GIS and accelerometer measurements were included in the analysis. We excluded 118 (25.70%) individuals without valid GIS and accelerometer data.

### Data collection procedure

The data collection followed baseline procedures developed in 2009. Participants were interviewed in the language of their choice. For anthropometrics, participants were invited to a convenient center (e.g. community school premises and church) where trained research assistants carried out all physical measurements and introduced accelerometers. A dedicated research assistant instructed and demonstrated how to wear the monitor above the right hip during all waking hours for seven consecutive days, except during water activities and showering. All protocol and standard guidelines were followed during the initializing period of the accelerometers [27]. Socio-demographic characteristics included age, gender, marital status, education level, employment status, family income, smoking, alcohol use and ownership of motor cars in the household.

### Anthropometric measures

Height to the nearest 0.1 cm was measured using a portable stadiometer with participant standing in bare feet and upright. Weight to the nearest kilogram, was measured using a portable bathroom weighing scale (Soehnle, Germany), with participants lightly dressed. BMI was calculated as weight (kg) divided by height (m^2^).

### Blood pressure measurement and definition of hypertension

Trained staff measured blood pressure (BP) using an Omron 711 (IT; Omron Healthcare Europe BV, Hoofddorp, The Netherlands) automated device with the appropriate cuff size for the measured mid-upper-arm circumference and taken after the subject had been seated at rest for at least 10 min. Blood pressure measurements were taken on the left arm of each participant in a sitting position. Two readings were made 3–4 min apart and the averaged reading used for the definition of hypertension (systolic blood pressure-SBP ≥ 140 mmHg and/or diastolic blood pressure-DBP ≥ 90 mmHg). In the current study, systolic and diastolic blood were treated as continuous variables. Additionally, self-reported hypertension was defined as previously diagnosed hypertension by a physician based on a positive answer to the question “*have you been diagnosed with hypertension?*”

### Physical activity

Objective measurement of physical activity (PA) was done using the ActiGraph GT3X (ActiGraph, Shlimar, FL, USA). The technical specifications and performance characteristics of the Actigraph accelerometer have been described elsewhere [28]. Actigraph data were considered complete if the participants had accelerometer counts for at least 10 h per day for at least 4 days. At least 30 min of continuous zero counts were regarded as accelerometer non-wear periods. The epoch length was set at 60-s epochs. We defined moderate physical activity (MPA), vigorous physical activity (VPA) and moderate- and vigorous-intensity activity (MVPA) cut-points for adults using previously published recommendations [29]. The study used the following cut-off points: MPA (2691–6166), VPA (6167–9642) and MVPA (≥9643) [29]. The data were scored and interpreted using the software program ActiLife version 6 (ActiLife software; Pensacola, FL; USA). Accelerometers have been recognized as a valid and objective tool to assess free-living physical activity [30].

### Built environment attributes

Built environment attributes were assessed using Global Information System (GIS) -derived variables. The location of each participant’s residence was geocoded using ArcGIS 9.3 (ESRI Inc). Three road distance buffers (500 m, 1000 m and 1600 m) were generated around each participant’s household [31]. Neighborhoods were defined by constructing a buffer zone around each participant’s home based on a street network [15]. Consequently, the study used road distance buffers that reflected walking times of approximately 5–7 min (500 m), 10–12 min (1000 m) and 15–18 min (1600 m) at a normal walking pace [32]. The study measured destination proximity to services and facilities such as the community center (defined as a complex that included a police station, health clinic and open space), distance to transit stops (taxi rank) and retail shopping center (shopping mall). GIS data were provided by the Faculty of Engineering and Built Environment from University of Cape Town.

#### Statistical analysis

The descriptive data are presented as means ± standard deviation or count and percentages. The Chi squared test and t-test were used to compare socio-demographic characteristics selected CVD risk factors and built environment attributes by gender. In age and sex adjusted models, linear regression analyses using 500 m as reference buffer were executed to assess the association between built environment attributes and selected CVD risk factors including BP, BMI and PA. Statistical tests were considered significant at *p* < 0.05. All data were analysed using SPSS® version 22 for Windows (IBM Corp: Armonk New York).

## Results

### Descriptive characteristics of the participants

The socio-demographic characteristics of the participants are displayed in Table 1. Of the 341 participants, 77.4% were female and the mean age was 56.1 ± 10.6 years than male 54.4 ± 11.9, *t* (339) = − 1.64, *p* = 0.101, *d* = 0.22. Furthermore, 41.9% of the participants were unmarried, 61.9% had completed secondary school, 61.9% were unemployed, 93.5% earned between ZAR2000–5000 per month and 92.7% did not own a car. These characteristics were similar between men and women (all *p* > 0.093). Additionally, 28.2% of the participants smoked while 35.2% were current drinkers, with significant gender differences in both smoking and drinking status (both *p* ≤ 0.005), Table 1.

**Table 1:** descriptive characteristic of the participants by sex

Variables	Male	Female	*P*-value	All
N (%)	77 (22.6)	264 (77.4)		341
Age (M^a^ ± SD)	54.4 ± 11.9	56.6 ± 10.2	0.101	56.1 ± 10.6
Socio-demographic (N (%))				
Marital status			0.093	
Never	29 (37.7)	114 (45.2)		143 (41.9)
Current	28 (36.4)	56 (21.2)		84 (24.6)
Co-habiting	5 (6.5)	20 (7.6)		25 (7.3)
Single	3 (3.9)	11 (4.2)		14 (4.1)
Widowed/divorced/separated	12 (15.6)	63 (23.9)		75 (22.0)
Education Level			0.581	
Primary	19 (24.7)	67 (25.4)		86 (25.2)
Secondary	46 (59.7)	165 (62.5)		211 (61.9)
Vocation	7 (9.1)	24 (9.1)		31 (9.1)
College/University	5 (6.5)	8 (3.0)		13 (3.8)
Employment Status			0.332	
Full time	10 (13.0)	21 (8.0)		31 (9.1)
Part time	7 (9.1)	14 (5.3)		21 (6.2)
Self employed	3 (3.9)	12 (4.5)		15 (4.4)
Unemployed	41 (53.2)	170 (64.4)		211 (61.9)
Retired	16 (20.8)	47 (17.8)		63 (18.5)
Salary levels ^b^			0.704	
≤ ZAR 2000	3 (3.9)	17 (6.4)		20 (5.9)
ZAR 2000–5000	72 (93.5)	240 (90.9)		312 (91.5)
≥ ZAR 5000	2 (2.6)	7 (2.7)		9 (2.6)
Smoking			**0.005**	
Never	29 (37.7)	155 (58.7)		184 (54.0)
Current	30 (39.0)	66 (25.0)		96 (28.2)
Former	18 (23.4)	43 (16.3)		61 (17.9)
Alcohol			**0.004**	
Never	22 (28.6)	122 (46.2)		144 (42.2)
Current	39 (50.6)	81 (30.7)		120 (35.2)
Former	16 (20.8)	61 (23.1)		77 (22.6)
Own a car			0.340	
None	74 (96.1)	242 (91.7)		316 (92.7)
One	1 (1.3)	13 (4.9)		14 (4.1)
Two or more	2 (2.6)	9 (3.4)		11 (3.2)
CVD risks - self-reported				
Hypertension			**0.004**	
No	53 (68.8)	133 (50.4)		186 (54.5)
Yes	24 (31.2)	131 (49.6)		155 (45.5)
CVD risks - screened (M^a^ ± SD)				
BMI (kg/m^2^)	26.3 ± 6.5	33.0 ± 6.8	**< 0.001**	31.5 ± 7.3
SBP (mm Hg)	139.3 ± 23.0	143.7 ± 22.6	0.126	142.7 ± 22.7
DBP (mm Hg)	81.5 ± 12.5	85.3 ± 12.5	**0.047**	84.6 ± 12.5
Accelerometer physical activity† (M^a^ ± SD)^c^				
Moderate (counts/minutes)	300.0 ± 111.8	314.3 ± 118.5	0.540	311.4 ± 116.9
Vigorous (counts/minutes)	3.9 ± 3.1	3.4 ± 3.5	0.509	3.5 ± 3.4
Total MVPA (counts/minutes)	303.9 ± 114.9	317.7 ± 122.0		314.9 ± 120.3
Objectively measured built environment (N (%)) m^d^				
Community center			0.835	
500	49 (63.6)	159 (60.2)		208 (61.0)
1000	12 (15.6)	48 (18.2)		60 (17.6)
1600	16 (20.8)	57 (21.6)		73 (21.4)
Shopping center			0.124	
500	28 (36.4)	118 (44.7)		146 (42.8)
1000	37 (48.1)	93 (35.2)		130 (38.1)
1600	12 (15.6)	53 (20.1)		65 (19.1)
Taxi Station			0.693	
500	39 (50.6)	147 (55.7)		169 (49.6)
1000	9 (11.7)	31 (11.7)		105 (30.8)
1600	29 (37.7)	86 (32.6)		67 (19.6)

*M*^*a*^ mean*, ZAR*^*b*^ South African Rand*, SD* standard deviation*, CVD* cardiovascular disease*;* ^*c*^subsample of 104*, m*^*d*^ meter

All bold entries are significant (*p* <0.05)

The mean BMI was higher in women (33.0 ± 6.8) than in men (26.3 ± 6.5), *t* (339) = − 7.69, *p* = < 0.001, *d* = 0.09, Table 1. The mean blood pressure level was 142.7 ± 22.7 mmHg (systolic) and 84.6 ± 12.5 mmHg (diastolic). Mean DBP was higher in female (85.3 ± 12.5) than in male (81.5 ± 12.6), *t* (339) = − 2.29, *p* = 0.047, *d* = 0.29.

The mean counts per minute spent in objectively measured MPA was 314.0 ± 116.9 for female and (300.0 ± 118.8) for male, *t* (102) = − 0.615, *p* = 0.540, *d* = 0.12. The mean MVPA was similar in males (3.9 ± 3.1) and females (3.4 ± 3.5), *t* (102) = 0.66, *p* = 0.509, *d* = 0.13. Total MVPA was also similar in females (314.9 ± 120.3) and males (309.3 ± 115.8), *t* (102) = − 0.61, *p* = 0.543, *d* = 0.12.

The GIS buffer from participants’ home was predominantly 500 m (61.0% of participants) for the community center, 500 m (42.8%) for the shopping center and 500 m (49.6%) for the taxi station. Likewise, no gender differences were noted across BE attributes (*p* > 0.05), Table 1.

### Associations of objectively assessed built environment attributes and selected CVD risk factors

In univariate linear regression models (Table 2), distance from a participant’s home to the community center was associated with higher diastolic blood pressure. Relative to 500 m, residing 1000 m away from the shopping center was associated with 2.31 (0.53 to 4.09) kg/m^2^ higher BMI and 4.51 (− 0.68 to 8.36) mmHg higher DBP, while residing 1600 m away was associated with 2.40 (0.14 to 4.57) kg/m^2^ higher BMI. Residing 1000 m away from the taxi rank was associated with 5.22 (1.52 to 8.88) mmHg lower DBP, Table 2.

**Table 2:** Univariable linear regression of objectively measured built environment attributes with selected CVD risk factors

Variables	BMI (kg/m^2^)	SBP (mmHg)	DBP (mmHg)	MPA (counts/minute)†	VPA (counts/minute)†	MVPA (counts/minute)†
	β (95% to CI)	β (95% to CI)	β (95% to CI)	β (95% to CI)	β (95% to CI)	β (95% to CI)
Objectively measured Built environment (m)						
Community center						
500 (ref)						
1000	0.70 (−1.81 to 3.21)	3.91 (−2.67 to 10.48)	4.02* (0.38 to 7.62)	−63.23 (− 127.18 to 0.73)	0.01 (−1.60 to 1.61)	31.44 (−24.42 to 87.30)
1600	1.84 (−0.28 to 3.95)	5.90 (− 2.12 to 13.51)	5.43* (1.13 to 9.73)	−26.07 (− 180.00 to 27.86)	−0.26 (− 1.66 to 1.45)	−40.25 (−88.99 to 8.48)
Shopping center						
500 (ref)						
1000	2.31* (0.53 to 4.09)	4.63 (− 0.74 to 10.09)	−4.51* (− 8.36 to 0.68)	23.23 (− 13.97 to 60.38)	− 0.01 (− 1.19 to 1.17)	−21.61 (−60.11 to 17.00)
1600	2.40* (0.14 to 4.57)	2.82 (− 4.01 to 9.62)	− 0.32 (−3.24 to 2.67)	− 43.30(− 89.34 to 2.76)	0.12 (− 1.58 to 1.83)	− 46.24 (−93.90 to 1.54)
Taxi Rank						
500 (ref)						
1000	−1.71 (−4.32 to 0.98)	−2.21 (− 10.28 to5.38)	−5.22** (− 8.88 to − 1.52)	− 30.40 (− 83.18 to 22.31)	0.40 (− 0.83 to 1.55)	−32.33 (− 87.00 to 22.38)
1600	−1.60 (− 4.12 to 0.92)	1.73 (− 3.67 to 7.02)	2.24 (− 0.81 to 5.14)	−42.34 (− 88.3 to 3.74)	− 0.30 (− 1.96 to 1.36)	−46.41 (− 94.06 to 1.35)

*BMI* body mass index*, SBP* systolic blood pressure*, DBP* diastolic blood pressure*, MPA* moderate physical activity*, VPA* vigorous physical activity*, MVPA* moderate vigorous physical activity*;* ***†*** subsample of 155; *β* beta coefficient*, CI* confidence interval; *m* meter; *p < 0.05; **p < 0.01

^†^Physical activity variables

In multivariate regression models, adjusted for sex and age, the distance to a community center (1000) remained associated with BMI 4.70 (2.06 to 7.34), DBP 4.97 (− 0.00 to 9.95) mmHg and MVPA -69.30 (− 134.92 to − 3.70). The distance to shopping center (1600 m) was positively associated with BMI [beta 4.78 (1.11 to 8.45)] and 1000 m was associated with SBP [76.99 (0.03 to 83.95)] (Table 3). In an extended multivariable regression model even after adjusting for history of hypertension, distance to a community center (1000 m) was positively associated with DBP 5.4 (0.4 to 10.3) mmHg. Similarly, shopping center (1000 m) remained positively associated with DBP 6.4 (1.4 to 11.4) mmHg, (Table not shown).

**Table 3:** Multivariable linear regressions of objectively assessed built environment with CVD risk factors adjusted for age and sex^a^

Variables	BMI (kg/m^2^)	SBP (mmHg)	DBP (mmHg)	MPA (counts/minute)†	VPA (counts/minute)†	MVPA (counts/minute)†
	β (95% to CI)	β (95% to CI)	β (95% to CI)	β (95% to CI)	β (95% to CI)	β (95% to CI)
Objectively measured built environment (m)						
Community center						
500 (ref)						
1000	4.70*** (2.06 to 7.34)	44.08 (−4.67 to 52.82)	4.97* (−0.00 to 9.95)	− 29.10 (−82.60 to 24.43)	0.0 (−1.60 to 1.64)	−34.56 (−90.00 to 20.90)
1600	2.37 (− 0.30 to 5.02)	6.21 (− 2.59 to 15.01)	6.58** (1.57 to 11.58)	−60.90 (− 124.25 to 2.44)	−0.22(− 2.13 to 1.70)	−69.30 *(− 134.92 to − 3.70)
Shopping center						
500 (ref)						
1000	1.79 (−0.32 to 3.89)	76.99* (0.03 to 83.95)	−1.82 (−5.79 to 2.14)	−61.50 (− 208.99 to 86.00)	−1.72 (− 10.19 to .75)	−60.79 (− 212.73 to 91.16)
1600	4.78** (1.11 to 8.45)	− 0.63 (− 9.51 to 8.26)	−0.68 (−7.59 to 6.22)	−127.22 (− 358.73 to 104.29)	1.50 (− 6.44 to 9.44)	−124.81 (− 363.00 to 113.68)
Taxi Rank						
500 (ref)						
1000	−0.11 (− 2.81 to 2.59)	0.67 (−8.26 to 9.61)	1.50 (− 3.59 to 6.58)	22.13 (−147.83 to 192.09)	−2.78 (− 11.78 to 6.22)	21.17 (− 153.92 to 196.26)
1600	− 1.68 (− 4.07 to 1.70)	7.71 (− 0.19 to 15.60)	3.35 (− 1.14 to 7.84)	102.31(− 57.00 to 261.61)	3.56 (− 2.23 to 9.35)	104.62 (−59.49 to 268.73)

*BMI* body mass index, *SBP* systolic blood pressure, *DBP* diastolic blood pressure, *BP* blood pressure, *MPA* moderate physical activity, *VPA* vigorous physical activity, *MVPA* moderate vigorous physical activity; ***†*** sample of 155; *β* beta coefficient; *p < 0.05; **p < 0.01; ***p < 0.001

^a^adjusted for age and sex

^†^Physical activity variables

## Discussion

In the current study, participants, in general, met the WHO recommendations of 150 min/week for MPA and MVPA, but with very low levels of VPA. In univariate regression analysis, we observed statistically significant positive relationships between objectively measured distances to the shopping center and body mass index. While, distance to a community center was positively associated with diastolic blood pressure, proximity to a shopping center was inversely associated with diastolic blood pressure. After adjusting for age and gender, the distance to a community center (1000) remained associated with BMI, DBP and MVPA. The distance to shopping center (1600 m and 1000 m) were positively associated with BMI and SBP, respectively (Table 3). In a further adjustment for history of hypertension, distance to a community center (1000 m) and shopping center (1000 m) remained positively associated with DBP.

This study supports the growing evidence that a walkable neighborhood environment is associated with fewer CVD risk factors [23, 33, 34]. For example, in a subjective assessment of neighborhood environment, attributes such as proximity to local stores, transit stops, four-way intersections, sidewalks, shade from trees, pleasant scenery, high traffic volume, crosswalks and well-lit streets were significantly associated with physical activity [35]. In a similar study, land use mix-diversity and safety from traffic was associated with hypertension [36]. Another study consistently found that built environment attributes were associated with obesity [37].

A number of potential advantages have been suggested that explain the beneficial effects of living near community services, including increased physical activity, and lowering obesity and hypertension [38]. However, the mechanism by which the interaction between built environment attributes and CVD risk factors occurs has yet to be fully elucidated. Nevertheless, one possible interpretation could be that the short distance from the respondents’ home to the community center, for example, increases physical activity through walking, thereby lowering the risk of developing obesity and hypertension. Considering the low wages of our participants and the low prevalence of car ownership, it is likely that most participants were relying on walking and public transport essentially, when they perceive proximal rather than distant destinations. Because individuals participate in regular physical activity, availability of facilities within walking distance can positively impact on energy expenditure which may assist in weight control [37].

Another potential mechanism for the relationship between proximity to a community center, and health outcomes may be found in a study from the Netherlands, where high quality, open public space was associated with a lower systolic blood pressure [38]. Another study showed that individuals living in sprawling areas are likely to walk less in their leisure time, overweight, and have greater prevalence of hypertension than those living in more compact places [39]. This implies public open space may affect health, not only through providing physical activity space, but also through its contribution to environmental improvement, such as air quality. In addition, it is possible that individuals living close to the community center (health facility) may be more aware of their hypertension status and able to access anti-hypertensive drugs. For example, in Ethiopia individuals from long distant areas were less likely to be adherent to hypertension treatment as compared to those who resided closer to health centers [40].

In this study setting, the shopping center was located near to the rail station, meaning that poor residents staying far away will usually need to take public transport to access the shopping center, thus resulting in less frequent walking trips. In addition, a proportion of our participants, who were employed, had to cover long distance to and from jobs. As a consequence, they may have had less time to shop and prepare healthy food for their households or may have had to depend on street food and ‘spaza’ shops for meals clustered near rail or bus stations, which were less unhealthy [41]. All these factors aggravate participants’ risk for health inequalities through unhealthy food choices and decreased physical activity, and subsequently favoring the development of cardio-metabolic risk factors such as obesity and hypertension.

### Limitation and strengths

The present study has some limitations. Its cross-sectional design did not allow defining causality association among characteristics. The study area, the Langa Township, has limited variability in land use, and consequently only variables available in the GIS database could be examined. For example, there was no GIS data available on local corner shops (spaza) considered to be selling unhealthy food. Therefore, more GIS data are required to better study BE association in South Africa. In addition, available variables were not categorized into contrasting socioeconomic levels (low/high) and built environments (low/high). Hence, these results are not necessarily being applicable to other neighborhoods in Cape Town. The study’s sample size may have been too small especially for accelerometer to observe clear differences and trends. Another concern is that although the accelerometers provide objective measures that remove self-report biases, they do not capture all forms of PA accurately (e.g. weight lifting, rowing, cycling). Moreover, participants were also asked to remove the monitors during activities involving water, which exclude swimming for instance from the PA data. Furthermore, the study didn’t measure muscle mass which has the potential to influence the BMI. The study also had strengths. This is the first study to provide evidence on the direct association between objectively measured built environment and physical activity with BMI, SBP, DBP, in an Africa context.

## Conclusion

The current study has provided for the first time, evidence to support an association between access to community resources and amenities, proximity to community and shopping centers, as well as transit stops, and objectively measured physical activity, along with CVD risk factors, BMI, SBP and DBP among South African adults. The current study indicates that public health approach such as increasing proximity and access of community services in neighborhoods would potentially assist in reducing population’s cardio-metabolic disease risk, by creating opportunities for physical activity and healthy food choices. However, similar studies should be replicated in other African countries before any firm conclusions can be ascertained.

## Abbreviations

BMI: Body Mass Index
BP: Blood pressure
CVD: Cardiovascular disease
DBP: Diastolic blood pressure
GIS: Geographic information system
MPA: Moderate physical activity
MVPA: Moderate-to-vigorous physical activity
PA: Physical activity
PURE: Prospective Urban Rural Epidemiology
SBP: Systolic blood pressure
SPSS: Statistical package for the social sciences
USA: United States of America
ZAR: South African Rand
